# Research Progress on the Role of Microglia Membrane Proteins or Receptors in Neuroinflammation and Degeneration

**DOI:** 10.3389/fncel.2022.831977

**Published:** 2022-02-25

**Authors:** Jun-Feng Zhao, Tong Ren, Xiang-Yu Li, Tian-Lin Guo, Chun-Hui Liu, Xun Wang

**Affiliations:** ^1^Department of Neurosurgery, Affiliated Dalian No. 3 People’s Hospital, Dalian Medical University, Dalian, China; ^2^Department of Neurosurgery, Beijing Tiantan Hospital, Beijing, China

**Keywords:** microglia membrane protein, receptor, neuroinflammation, novel coronavirus, review

## Abstract

Microglia are intrinsic immune cells of the central nervous system and play a dual role (pro-inflammatory and anti-inflammatory) in the homeostasis of the nervous system. Neuroinflammation mediated by microglia serves as an important stage of ischemic hypoxic brain injury, cerebral hemorrhage disease, neurodegeneration and neurotumor of the nervous system and is present through the whole course of these diseases. Microglial membrane protein or receptor is the basis of mediating microglia to play the inflammatory role and they have been found to be upregulated by recognizing associated ligands or sensing changes in the nervous system microenvironment. They can then allosterically activate the downstream signal transduction and produce a series of complex cascade reactions that can activate microglia, promote microglia chemotactic migration and stimulate the release of proinflammatory factor such as TNF-α, IL-β to effectively damage the nervous system and cause apoptosis of neurons. In this paper, several representative membrane proteins or receptors present on the surface of microglia are systematically reviewed and information about their structures, functions and specific roles in one or more neurological diseases. And on this basis, some prospects for the treatment of novel coronavirus neurological complications are presented.

## Introduction

**FIGURE 1:**
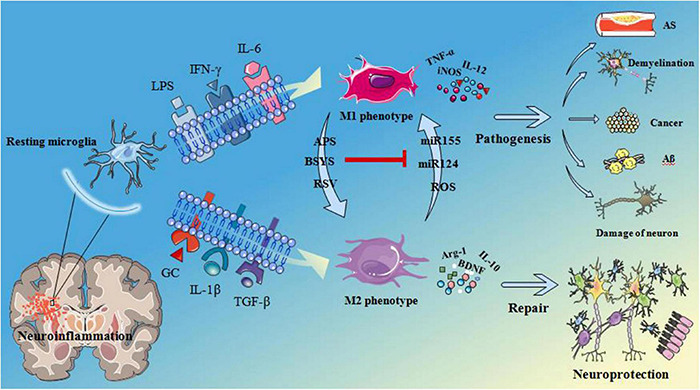
Microglia are activated as M1 phenotype when microglia surface membrane proteins or receptors recognize LPS, INF-γ, IL-6. Then release TNF-α, inducible nitric oxide synthase (iNOS), IL-12 and other pro-inflammatory factors leading to atherosclerosis (AS), demyelination, cancer, beta amyloid (Aβ) deposition and fibrillary tangles and neuronal damage. When microglia surface membrane proteins or receptors recognize glucocorticoid (GC), IL-1β, TGF-β, etc. Microglia are activated as M2 phenotype and release arginase-1 (Arg-1), brain derived neurotrophic factor (BDNF), IL-10 these pro-inflammatory factors, play a neuroprotective role in nervous system. Astragalus polysaccharides (APS), Bu Shen Yi Sui capsule (BSYS) and resveratrol (RSV) can induce the transformation of M1 microglia into M2 microglia, On the contrary, micro ribonucleic acid 155 (miR155), miR124, and reactive oxygen species (ROS) can induce the transformation of M2 phenotype into M1 phenotype. APS, etc., can inhibit the effects of miR155 these substances that is, inhibit the transformation of microglia from M1 phenotype to M2 phenotype.

Neuroinflammation acts as a key factor in the pathogenesis of a variety of central nervous system diseases, including neurodegenerative diseases and brain tumors, whereas the glial cells play a pivotal role in the regulation of neuroinflammation. The various membrane proteins or receptors, such as translocation protein 18 kDa (TSPO), protease-activated receptor 1/4, receptors for advanced glycation end products, etc., have been found to influence neuroinflammation, and provide an important link in the nervous system injury and repair, neurodegeneration, and nervous system tumors, by effectively modulating microglia activation. So these proteins or receptors may serve as important therapeutic targets to treat various neurodegenerative diseases and inhibit tumor growth. Therefore, regulation of the membrane proteins and their downstream proteins in glial cells is a challenging but promising approach to suppress neuroinflammation and treat a variety of neurological diseases, including Alzheimer’s disease (AD), Parkinson’s disease (PD), amyotrophic lateral sclerosis (ALS), and glioma. However, there are only few systematic studies on microglia membrane proteins or receptors and there are also few relevant literatures describing their functions. Therefore, the authors have evaluated the various articles from the perspective of time and correlation degree, and compiled a review on the role of different microglia membrane proteins and their functions in mediating neuroinflammation in various neurological diseases. Microglia, the resident macrophages in the central nervous system, express receptors for the classical neurotransmitters (Logiacco et al., 2021), was the first and most important immune line identified in the central nervous system (CNS). As resident immune effector cells in CNS, microglias and their mediated neuroinflammation can play a very important role in the process of CNS injury and disease progression. Microglia have been found to be sensitive to CNS injury and can rapidly proliferate, increase or re-express histocompatibility complex (MHC) antigen, migrate and transform into phagocyte-like morphology (amoeba-like), In addition, they can at the same time secrete a large number of different cytokines and cytotoxic substances such as liposolysaccharide (LPS), Interferon-γ (IFN-γ), interleukin-6 (IL-6), etc. At the late stage of inflammation caused by injury, various neurotrophic factors such as brain-derived neurotrophic factor (BDNF) are mainly secreted, which can provide optimal nutrition and facilitate the repair of neurons. Microglia could be activated into two different states, classic activated state (M1 state) and alternative activated state (M2 state). It has been established that and M1 state is harmful, but M2 is beneficial (Guo et al., 2021). In addition, M1 microglia can contribute to the development of inflammation upregulating pro-inflammatory cytokines, while M2 microglia can exert anti-inflammation effects through significantly enhancing the expression of the various anti-inflammatory factors. Moreover, M1 and M2 microglia could be mutually transformed into one another under various conditions (Wang J. et al., 2021; Figure 1). When exposed to cytokines such as Tumor necrosis factor-α (TNF-α) or IFN-γ, microglia are activated and exhibit an M1-like phenotype, generating enzymes and reactive oxygen species (ROS) that can effectively promote persistent tissue inflammation through the production of inflammatory cytokines, resulting in a harmful neuronal microenvironment (Liu et al., 2016). When exposed to interleukin-4 (IL-4), interleukin-1β (IL-1β), glucocorticoids, transforming growth factor-β (TGF-β), or interleukin-10 (IL-10), microglia can differentiate into an anti-inflammatory M2 phenotype (Honjoh et al., 2019), thereby secreting the various neurotrophic factors and anti-inflammatory mediators that induce the production of a supportive neuronal microenvironment that can facilitate to eliminate inflammation and repair the damage tissues (Orihuela et al., 2016; Kim and Son, 2021). In addition, disease-related molecular patterns, such as TNF-α and IFN-γ, have corresponding membrane proteins or receptors on the surface of microglia, and these also produce different effects after interacting with their corresponding ligands (Hartmann et al., 2015), such as maintaining the bipolarity of microglia in the nervous system. In this paper, we have described the various microglia cell membrane proteins or receptors that can primarily produce M1 effect after the recognition of corresponding ligands such as IFN-γ and LPS, and highlighted the role of neuroinflammation mediated by them in nervous system injury and degeneration.

## Translocation Protein 18 kDa

Translocation protein 18 kDa was initially identified as the first subunit of peripheral type benzodiazepine receptor (PBR), that can function as diazepam binding inhibitor located in the outer membrane of mitochondria (DBI), and was renamed translocator protein 18 kDa (TSPO) in 2005. In the CNS, it is mainly distributed in the mitochondrial outer membrane of glia cells (Lee Y. et al., 2020), expressed in resting microglia, and significantly upregulated when microglia are activated (Zhang et al., 2021). There are voltage-dependent anion channels (VDAC) and adenine nucleoside *trans*-locase (ANT) that can directly bind to benzodiazepines (Barresi et al., 2021). These three together constitute the TSPO receptor complex VDAC and ANT (Marginedas-Freixa et al., 2018) are involved in the formation of mitochondrial permeability transition pore (MPTP), which has been related to cell apoptosis (Kołodziejczyk, 2015). In addition, TSPO and its complexes have been found to exhibit various physiological functions (Betlazar et al., 2020), such as promoting the production of neurosteroids, regulating mitochondrial respiration apoptosis, cellular immunity, cell growth and proliferation (Shoshan-Barmatz et al., 2019). TSPO can also effectively bind with different ligands as a receptors, endogenous ligands include diazepam binding inhibitor (DBI), porphyrins, etc., (Kołodziejczyk, 2015). Exogenous ligands including 1-(2-Chlorophenyl)-*N*-methyl-*N*-(1-methylpropyl)-3-isoquinolinecarboxamide (PKlll95) and 4′-chlorodiazepam (R05-4864) (Keskin et al., 2021; Yusuying et al., 2021) can bind to TSPO receptor can modulate its diverse physiological function, such as modulation of neurosteroid synthesis, etc. TSPO is an important component of the steroid synthesis (Selvaraj et al., 2015). It can promote the transmembrane transport of the cholesterol into the phospholipid membrane and thereby increase the formation of pregnenolone to promote the downstream neurosteroid synthesis (Lejri et al., 2019). It has an important role in repairing of the damaged brain nerves and promoting nerve growth (Kim et al., 2021). TSPO imaging agent can be effectively used for nerve mental disease diagnosis and its expression in the damaged brain tissue has been found to be significantly higher than that of normal brain tissue expression, Thus, It can be potentially used as an biomarker of brain injury and neural degenerative diseases, which can observe TSPO by PET and SPECT with the combination of the ligands and the brain damage under the condition of TSPO regulation changes (Ji and Xu, 2021). TSPO has been reported to be involved in the inflammatory response of CNS and can contribute to the repair process of peripheral nerve injury. In addition, results of Sucksdorff et al. (2020) have strongly suggested that innate immune cell activation can substantially contribute to the diffuse neural damage leading to multiple sclerosis disease progression independent of relapses. Microglia-mediated chronic inflammation in the brain is one of the important early pathological features of neurodegenerative diseases (Guo and Zhu, 2021; Teipel et al., 2022). What’s more, activation and proliferation of microglia and astrocytes can lead to an increased production of the various inflammatory cytokines (García-García et al., 2021), such as IL-L, IL-6, interleukin-8 (IL-8), TNF-α and inducible nitric oxide synthase (iNOS), which can cause inflammation in the brain (Zhao et al., 2021). Ro5-4864 and PK11195, they are Peripheral benzodiazepine receptors agonist and antagonist, respectively. And after administration of them, the activity of microglia as observed to be decreased, and the levels of TNF-α, IL-1β and IL-6 secreted by microglia was also attenuated significantly (Choi et al., 2011). R05-4864 can also promote the survival and regeneration of neonatal motor neurons in adult rats (Veiga et al., 2005). Furthermore, the specific TSP0 ligand XBDl73 has also exhibited rapid anti-anxiety effect (Bloms-Funke et al., 2021; Rupprecht et al., 2021).

## Myeloid Triggering Receptor 2

The triggering receptor expressed specifically on myeloid cells 2 (TREM2) present on the surface of microglia is a member of the immunoglobulin superfamily and a transmembrane cellular immunomodulatory receptor (Ruganzu et al., 2021). It is involved in the proliferation, survival, migration, phagocytosis and inflammatory regulation of microglia (Xie et al., 2021). TREM2 is primarily a one-way transmembrane receptor that consists of an extracellular V-type immunoglobulin (Ig) domain, a transmembrane region of lysine residues, and a short cytoplasmic tail which without any function about transduction activation signaling. In brain tissue, TREM2 is expressed only in microglia (Sheng et al., 2021). It has been found that binding of TREM2 to DNAX-activating protein of 12 kDa (DAPl2) can mediates the downstream signal transduction that can affects microglia function (Konishi and Kiyama, 2020). Moreover, recent studies have found that TREM2 can increase AD risk by about 3-times (Roussos et al., 2015). It was observed that during early stage of AD, the major changes in microglia were characterized by upregulation of TREM2/DAP12, phagocytosis-related genes, and anti-inflammatory genes. Elevated expression of TREM2/Nuclear factor-kappa B (NF-κB) and proinflammatory genes were observed at the late stage (Ji et al., 2021). TREM2 can recognize misfolding, apoptotic cells and the dead neurons (Takahashi et al., 2005; Kawabori et al., 2015; Kurisu et al., 2019). TREM2 has been shown to modulate microglia to enhance their phagocytic function and inhibit microglia proinflammatory effects, thus providing neuroprotection against ischemic brain injury. Zhai et al. (2017) demonstrated that TREM2 may be involved in polarization of microglia phenotype by microglia microphenotype cell trigger assay. In addition, upregulation of TREM2 can promote M2-type polarization of microglia, inhibit inflammation, and markedly promote the recovery of neurological function after ischemia (Zhai et al., 2017). Zhang et al. (2018) reached the same conclusion from experimental study on microglia chronic virus infection, thereby reducing the inflammatory response involved in the pathological process of PD. In ischemic brain injury, TREM2 negatively regulates Toll-like receptor 4 (TLR4)-mediated inflammation by inhibiting Extracellular signal-regulated kinase (ERK) phosphorylation (Whittaker et al., 2010), binding to adaptor protein 12 kDa DNAx (DAPl2) (Peng et al., 2013) and thus reducing NF-κB activation (Rosciszewski et al., 2018). Furthermore, TREM2 can induces microglia activation as well as chemotaxis, and absence of TREM2 has been found to inhibit microglia proliferation (Zheng et al., 2016). Moreover Mazaheri et al. (2017) demonstrated that loss of TREM2 impaired microglia response to injury and attenuated chemotaxis signaling, thereby reducing microglia migration to ischemic lesions and apoptotic neurons. In AD, the ability of microglia to prune Aβ plaques has been found to be impaired, changes in the plaque compaction occur, the levels of neurotoxic oligomic Aβ as well as fibrous Aβ increase, thus leading to neuronal malnutrition and death (Mazaheri et al., 2017). Due to substantial plaque compaction changes, microglia are less capable of phagocytosis and hence unable to eliminate Aβ and apoptotic cells (Poliani et al., 2015; Yuan et al., 2016), thereby adversely affecting the AD process.

## Receptors for Advanced Glycation End Products

The receptor for Advanced Gly-cation end products (RAGE) is a characteristic cell surface receptor of advanced gly-cation end products, which belongs to the immunoglobulin family (Derk et al., 2018; Wang Q. et al., 2021). RAGE can serve as a pro-inflammatory pattern recognition receptor that is primarily expressed on microglia and astrocytes. It plays an important role in inflammation, oxidative stress and cellular dysfunction in a number of neurodegenerative diseases including AD, PD and ALS (Lee J. et al., 2020). There are numerous RAGE ligands that can exhibit a wide range of functions. Activation of RAGE can promote intracellular oxidative stress, regulate the activities of different protein kinases and nuclear factors, and promote the occurrence of various neurological diseases and cardiovascular diseases, as well as the growth, invasion and metastasis of tumors (Jangde et al., 2020; Leerach et al., 2021). RAGE is a complete membrane protein which composed of three distinct parts: a large extracellular domain, a transmembrane domain and a short intracellular domain. There were 3 immunoglobulin-like regions (1 V region and 2 C region) in the extracellular domain of RAGE, which have been found to be important for the recognition of the specific ligands. Its intracellular domain is small, rich in charge, and can bind to the various intracellular signaling molecules, which are related to the signal transduction of RAGE (Syed et al., 2018). Due to the selective splicing of their mRNA, three different kinds of allotropes have been identified: N-terminal truncated RAGE, C-terminal truncated RAGE (sRAGE) and dominant negative RAGE (DN-RAGE) (Chellappa et al., 2021). The former can generally bind to the ligand, while the latter two can attach to the ligand without signal transduction, but can compete with the full length of RAGE. Therefore, the function of RAGE can be indirectly regulated by changing the ratio of different alloforms of RAGE (Xue et al., 2016). RAGE is mainly known for its role in (AD) (Ullah et al., 2020). The various receptors of RAGE can accelerate amyloidopathy by transporting Aβ across the blood brain barrier (BBB) and upregulating β-secretase and γ-secretase activities (Fang et al., 2018; Lee H. et al., 2021). Cytotoxic Aβ plaque deposition can significantly contributes to the pathological progression of AD (Chen et al., 2017), Aβ stimulates the secretion and the synthesis of RAGE ligand by functionally activating microglia, Aβ-RAGE interaction in the neurons thereby leading to the cell stress, ROS generation, and Aβ intraneuronal transport, which can cause mitochondrial dysfunction stimulate the various cell signaling pathways, such as mitogen-activated protein kinase (MAPK) pathway (Son et al., 2017). RAGE-dependent microglial activation, nuclear translocation of nuclear factor kappaB p65 (NF-κB p65), and the expression of downstream inflammatory mediators such as TNF-α, IL-1β, cyclooxygenase 2 (COX-2)/prostaglandin E2 (PGE2) and inducible nitric oxide synthase (iNOS)/nitric oxide (NO) (Shen et al., 2017). AGEs/RAGE interaction can significantly induce the release proinflammatory cytokines such as TNF-α, IL-6, IL-1 and iNOS in microglia (Berbaum et al., 2008). Overexpression of RAGE in microglia increases the tissue infiltration of glial cells, microglia activation, Aβ accumulation and deterioration of the various cognitive functions (Lv et al., 2014; Yu and Ye, 2015). In addition, M-CSF can bind with its receptor Cellular oncogene fos C-FOS on microglia to enhance the expression of RAGE mRNA, thus leading to the continuous activation of microglia in a positive feedback way, and thereby promoting the occurrence of long-lasting chronic neuroinflammatory reactions (Lue et al., 2001). RAGE has also been found to contribute to the stroke pathology through causing an upregulation of the various inflammatory cytokines (Cai et al., 2016). For example, in ischemic stroke, the necrotic core is surrounded by different areas of inflammation in which delayed cell death can exacerbate the initial damage. RAGE ligand high mobility group protein B1 (HMGB1) has been reported to be elevated in the serum of stroke patients, and RAGE receptors can act as sensors for the necrotic cell death, leading to inflammation and ischemic brain injury (Richard et al., 2017). it can also lead to delayed neuronal death after global cerebral ischemia by enhancing vascular injury and harmful glia-mediated inflammation (Menini et al., 2014; Lei et al., 2015). In addition, RAGE signaling in glioma-associated microglias and TAM can also substantially up-regulate vascular vascular growth factor (VEGF) in the tumor microenvironment (TME) to drive angiogenesis, and increase tumor-associated inflammation to promote tumor progression and invasion (Chen et al., 2014; Ha et al., 2021).

## Protease Activated Receptor 1/4

Protease-activated receptors (PARs) comprise a family of four G protein-coupled receptors (PAR1-PAR4) that are activated by the serine proteases derived from the coagulation cascade, including factor (F) Xa and thrombin (FIIa), immune cells, and pathogens (Willis Fox and Preston, 2020; Han et al., 2021). Protease-activated receptors are considered as non-classical pattern-recognition receptors (Friebel et al., 2021). PAR-3 and PAR-4 were initially thought to be receptors for thrombin and involved in thrombin signaling, while PAR-2 can be activated by trypsin and trypsin-like enzymes (Ocak et al., 2020; Pompili et al., 2021). PARS can effectively mediate the extracellar signal-regulated kinase (ERK1/2) signal transduction pathway to induce the nuclear reaction and activate a variety of cellular transcription factors (Yoon et al., 2017). Its physiological functions include inducing coagulation response, promoting cell division and proliferation, releasing inflammatory mediators or cytokines to regulate the local inflammatory response, and regulating vascular tension (Chandrabalan and Ramachandran, 2021; Lucena and McDougall, 2021). Activation of PARs can also stimulate cytoplasmic the phospholipase C, phospholipase A, phospholipase D, protein kinase C, mitogen-activated protein kinase (MAPK) and tyrosine protein kinase, temporarily increased cytoplasmic free calcium concentration, opened cell membrane ion channels, and promoted cellular growth (Aldrovandi et al., 2017; Thibeault et al., 2020).

Protease-activated receptors can be expressed in various cells of the nervous system, and its expression in microglia has been closely related to the activation of microglia and the production of inflammatory factors (Bushell et al., 2016). In particular, PAR1 and PAR4 play a pivotal role as thrombin receptors in the various hemorrhagic diseases of the nervous system such as cerebral hemorrhage (ICH) and subarachnoid hemorrhage (SAH) (Ye et al., 2021). Activated microglia after intracerebral hemorrhage can directly secrete matrix metalloproteinase (MMP), MMP-2 and MMP-3, which can significantly damage the blood-brain barrier (Zhu et al., 2016; Yang et al., 2021). At the same time, TNF-α and other inflammatory factors can be released to induce the activation of MMP-9 to promote the degradation the vascular wall matrix, and increase the permeability of BBB (Müller et al., 2021). The level of thrombin (the ligand of PARs) has been found to be elevated and the expression of PAR-1 and PAR-4 on the activated MG surface was highly consistent with the activation of MG (Wan et al., 2016) also reached the same conclusion, that PAR-1 was predominantly involved in ICH induced brain injury and regulate microglia polarization. It was found that M1 phenotype microglia increased significantly, reaching the peak at 4 h as early as ipthalateral basal ganglia, remaining high at 3 days, and decreasing at 7 days after ICH. Moreover, activation of M2 phenotype microglia/macrophages was delayed and peaked on day 1. Experimental animals treated with PAR-1 and PAR-4 small interfering RNA (siRNA) or PAR-1 deficient mice displayed substantial less M1 phenotype microglial activation, expression of MMP-9 and pro-inflammatory cytokines, inflammation, DNA damage as well as the neuronal death, brain swelling, and exhibited significant recovery of neurological function after ICH. These findings further illustrated the prominence of PAR-1 and PAR-4 mediated microglial activation to neurovascular injury (Wan et al., 2016). Thrombin, hemoglobin, plasma protein, C-reactive protein and other active substances are released by hematoma degradation after ICH, and thrombin plays an important role in the secondary brain injury after ICH (Babu et al., 2012; Kearns et al., 2021). It has been established that activation of PAR-1 is most likely caused by thrombin, and the expression of PAR-1 and PAR-4 was significantly upregulated, which may mediate a series of thrombin reactions, leading to the neurocytotoxic damage and death (Wan et al., 2016), In addition, thrombin may also affect aquaporin 4 function *via* PAR-1 mediated aggravation of the cerebral edema after ICH (Wu et al., 2010; Gao et al., 2015). Thus, targeting PAR-1, PAR-4 and their downstream interacting proteins may provide new opportunities to modulate the microglia-mediated inflammatory damage and recovery.

## CC Chemokine 2 (CCL2) Receptor (CCR-2)

CC Chemokine 2 (CCL2) namely Monocyte chemotactic protein-1 (MCP-1) and highly expressed in the cerebrospinal fluid of human multiple sclerosis (MS) patients (Stampanoni Bassi et al., 2021), and plays a key role in regulating in the migration of monocytes to the CNS (Kaushansky et al., 2019). It is a kind of small molecule protein that can display chemotactic action in response to the various immune cells, cause chemotactic inflammation. It can effectively cause aggregation of the neutrophils, monocytes, lymphocytes and other immune cells to the lesion site (Yu et al., 2020). It can also induce the synthesis of various cytokines such as IL-2, IL-6, and different cell adhesion molecules. As a kind of proinflammatory chemokine, CCL2 can effectively regulate the chemotaxis of monocyte derived macrophages, T lymphocytes, and dendritic cells to mediate neuroinflammation in the CNS. CC Chemokine 2 (CCL2) receptor also called as CC chemokine receptor 2 (CCR2), and CCR2 is the representative receptor of CC type chemokine. It has been shown that the CCL2-CCR2 axis can potentially activate microglia and influence the secretion of various proinflammatory factors such as IL-1β and interleukin-18 (IL-18) (Curzytek and Leśkiewicz, 2021). For instance, binding of CCL2 to CCR2 can activate the various intracellular signal transduction pathways, thereby leading to a series of intracellular changes related to cell migration or activation, including adhesion molecule upregulation or activation, receptor desensitization or internalized nuclear cytoskeletal rearrangement (Lee J. et al., 2021). It has been postulated that chemokines and their receptors play a pivotal role in the CNS for stimulating the migration of microglia, astrocytes, neurons, and neural stem cells as well as infiltrating immune cells during neuroinflammation (Gschwandtner et al., 2019). The role of CCL2 in the activation of microglia and astrocytes in neuroinflammation is well documented in literature (Ślusarczyk et al., 2015; Liddelow and Barres, 2017). CCR2 expression is significantly increased when the brain tissue is injured, and rapid desensitization of CCR2 is very important for accurate migration of the white blood cells to the lesion (Flynn et al., 2003). CCR2 can directly inhibit the activity of adenylate cyclase (Preobrazhensky et al., 2000). The decrease of intracellular cyclic adenosine phosphate cAMP level can also mediate various intracellular signaling cascades through modulating the JAK/STAT pathway (Follin-Arbelet et al., 2013). CCR2 may also activate protein kinase C by activating phospholipase C, stimulating the production of glycerol diester and Inositol triphosphate (IP3), thereby increasing the intracellular Ca2 + level and activating itself (Nardelli et al., 1999).

CCR2 has been closely correlated with hypoxic ischemic brain injury (HIBD). For instance, ischemia and hypoxia of the brain cells can significantly increase the expression of CCR2. After the cells expressing CCR2 migrate to the site of injury under the chemotaxis actions of CCL2, they bind with CCL2 to inhibit the migration of too many mononuclear macrophages, thus forming a self-limiting negative feedback loop (Loenen et al., 2001). The combination of CCL2 and CCR2 can also induce immune escape of the virus. For example, cytomegalovirus (HCMV) can encodes US28, which is homologous with CCR2. CCL2 can integrate into the host genes, causing the host cells to abnormally phagocytose chemokines, antagonize microglia activity, and regulate the migration of microglia. Thus inhibiting the chemokines around virus infected cells and preventing the aggregation of microglia can serve as useful strategy. CCR2 is involved in the chemotaxis and recruitment of the microglia in neurodemyelinating diseases such as autoimmune encephalomyelitis and MS (Huang et al., 2001). The combination of CCR2 and MCP-1 can further induce the recruitment of chemotactic microglia and lymphocytes in the endothelium of cerebral arteries and thereby enter the intima to become foam cells, which can participate in and aggravate cerebral atherosclerosis (Linton and Fazio, 2003). At the same time, the immune effect of the expression of CCR2 and its combination with CCL2 has been related to the occurrence of epilepsy and the formation of new blood vessels in (AD) (Grammas and Ovase, 2001).

## Discussion

In summary, these membrane proteins, which are specifically expressed on the surface of microglia and are also widely expressed in other cells, play an important role in causing the activation of microglia into a pro-inflammatory state and thus can effectively mediate neuroinflammation. Some of them activate microglia by recognizing disease related molecular patterns (PAMP) such as LPS and CPG-DNA, and others by sensing changes in the nervous system microenvironment such as ischemia, hypoxia, inflammatory factors, thrombin, and alterations in ion concentrations that are released after bleeding, etc. Further activation of microglia is thus achieved. Neurodegenerative changes are often the result of acute and chronic inflammation of the nervous system.

However, most of the studies on these membrane proteins primarily focus on the structure, function, distribution, and identifiable ligand of the protein or receptor, as well as the role of activating microglia mediated neuroinflammation itself or the synergistic effect of different proteins involved at the same level. How can these proteins or receptors, after recognizing PAMP and sensing changes in the nervous system microenvironment, continue to activate the various downstream signal transduction, transmit the recognized information to the nucleus, promote gene integration, and then get transcribed, translated and expressed as new proteins, or cause potential changes in the microglia cell intracellular environment is not clear. And the process that ultimately activates microglia and mediates neuroinflammation is not well described too. If we can consider these membrane proteins or receptors as potential targets, reduce microglial activation and the conversion from M2 anti-inflammatory to M1 pro-inflammatory by competitive inhibition of ligand analogs or direct silencing of receptors. On the other hand, after CCL2 binds to the ligand, blocks a certain link of its downstream signal transduction, and also inhibits the activation of microglia. Thus strategies to induce reduction or delay in the occurrence and process of neuroinflammation and neurodegeneration can be an important area to explore in the future.

With the development of research, people will have a more comprehensive and in-depth understanding of the receptors, released factors and involved signaling pathways during the activation of microglia. For instance, we can interfere some receptors expression effectively to reduce the activation of microglia, attenuate activation of inflammatory signaling pathway and the expression of proinflammatory cytokines or other harmful factors, in order to reduce significant damage to the nervous system (Luo et al., 2018; Liu et al., 2021). On the other hand, by up regulating the expression of M2 phenotype microglia or transforming microglia from M1 phenotype to M2 phenotype, the expression of anti-inflammatory cytokines can be enhanced. Furthermore, the phagocytosis and clearance of cell debris and Aβ can be facilitated to reduce the occurrence of the various neurodegenerative diseases.

Meanwhile, the national situation for COVID-19 remains alarming, as this virus is known to cause severe respiratory and circulatory symptoms (Li et al., 2020; Pascarella et al., 2020). The patients infected with COVID-19 can also display various neurological symptoms such as headache, dizziness, hypoesthesia and neuralgia and exhibit a series of complications (encephalopathy, acute cerebrovascular disorder and skeletal muscle injury) (Helms et al., 2020; Mao et al., 2020), that might need more and more attention. SARS-CoV-2 genetic material has been reported to be detected in cerebrospinal fluid (CSF) (Jakhmola et al., 2020). However, it is not clear whether the virus can invade the nervous system directly to produce an immune response or does it enter the nervous system through the damaged blood-brain barrier on the basis of damage to the respiratory and circulatory system and multiple organs and organs. There are three distinct ways through which the virus can potentially invade the nervous system and directly affect the brain: (a) Direct involvement, spread through the ethmoid plate during infection can lead to cerebral invasion (Netland et al., 2008). (b) The virus may use the neural pathways and enter the brain through the olfactory bulb (Payus et al., 2020). (c) The virus uses Angiotensin converting enzyme 2 (ACE2) receptor to enter the nervous system through the blood circulation pathway (Yan et al., 2020) and the expression of ACE2 receptor in brain glial cells and neurons (Baig et al., 2020; Vallamkondu et al., 2020).

However, it is not clear whether the novel coronavirus capture and cause phagocytosis of CCL2 secreted by virus-infected cells as human cytomegalovirus does, thereby preventing CCL2 from binding to the CCR2 on microglias, and thus hampering microglias from accumulating and performing immune functions, thereby achieving immune escape and ultimately causing further damage to the nervous system. In addition, it is not known if this escape function could be effectively suppressed and if the virus exposed to nervous system immune cells, such as microglia, could be affected to alleviate the various neurological symptoms. In this direction, we can further explore the detailed mechanism about how novel coronavirus lurks in the nervous system and causes nervous system damage. Such novel findings might aid to reduce its impact on the nervous system as well as mitigate the various complications, and speed up the recovery of pneumonia patients. Overall, additional studies are needed to provide new ideas for the prevention, control and treatment of COVID-19 worldwide, and improve the survival rate of COVID-19 patients.

## Author Contributions

XW and C-HL initiated the work and designed the idea. J-FZ, TR, T-LG, and X-YL prepared and collected material and data. J-FZ, TR, and X-YL wrote the manuscript. All authors reviewed the article, read and approved the submitted version.

## Conflict of Interest

The authors declare that the research was conducted in the absence of any commercial or financial relationships that could be construed as a potential conflict of interest.

## Publisher’s Note

All claims expressed in this article are solely those of the authors and do not necessarily represent those of their affiliated organizations, or those of the publisher, the editors and the reviewers. Any product that may be evaluated in this article, or claim that may be made by its manufacturer, is not guaranteed or endorsed by the publisher.
